# The Role of Structure and Biophysical Properties in the Pleiotropic Effects of Statins

**DOI:** 10.3390/ijms21228745

**Published:** 2020-11-19

**Authors:** Christopher Murphy, Evelyne Deplazes, Charles G. Cranfield, Alvaro Garcia

**Affiliations:** School of Life Sciences, University of Technology Sydney, Ultimo, NSW 2007, Australia; christopher.murphy@student.uts.edu.au (C.M.); evelyne.deplazes@uts.edu.au (E.D.); charles.cranfield@uts.edu.au (C.G.C.)

**Keywords:** statins, membranes, pleiotropic effects, hepatoselectivity, drug-membrane interactions

## Abstract

Statins are a class of drugs used to lower low-density lipoprotein cholesterol and are amongst the most prescribed medications worldwide. Most statins work as a competitive inhibitor of 3-hydroxy-3-methyl-glutaryl-coenzyme A reductase (HMGR), but statin intolerance from pleiotropic effects have been proposed to arise from non-specific binding due to poor enzyme-ligand sensitivity. Yet, research into the physicochemical properties of statins, and their interactions with off-target sites, has not progressed much over the past few decades. Here, we present a concise perspective on the role of statins in lowering serum cholesterol levels, and how their reported interactions with phospholipid membranes offer a crucial insight into the mechanism of some of the more commonly observed pleiotropic effects of statin administration. Lipophilicity, which governs hepatoselectivity, is directly related to the molecular structure of statins, which dictates interaction with and transport through membranes. The structure of statins is therefore a clinically important consideration in the treatment of hypercholesterolaemia. This review integrates the recent biophysical studies of statins with the literature on the physiological effects and provides new insights into the mechanistic cause of statin pleiotropy, and prospective means of understanding the cholesterol-independent effects of statins.

## 1. Introduction

Natural statins are fungal secondary metabolites produced in the polyketide pathway [1]. The first statin, compactin, was discovered in 1973, by Japanese biochemist Akira Endo, from culture broths of *Penicillium citrinum* [2,3,4,5,6,7,8]. The reason for the natural production of statins remains unknown, but it is speculated to be used against competing organisms [9,10,11,12,13]. Lovastatin, the first-ever commercial statin, was derived from *Monascus ruber* and *Aspergillus terreus* [2,3].

Clinically, statins are a class of amphiphilic compounds and are one of the most commonly prescribed drugs in the world used to treat hypercholesterolaemia [14,15]. The primary molecular mechanism of most statins is the competitive inhibition of 3-hydroxy-3-methyl-glutaryl-coenzyme A (HMG-CoA) reductase (HMGR), an enzyme involved in the rate-limiting step in cholesterol biosynthesis in hepatocytes [16]. Reduction in cellular cholesterol concentrations stimulates the cell-surface expression of low-density lipoprotein (LDL) receptors on hepatocytes, triggering the removal of circulating LDL cholesterol [17,18,19]. Moreover, this results in the increased concentration of the beneficial high-density lipoprotein cholesterol and lowered serum triglycerides [20,21]. Statins contain an HMG-CoA-like pharmacophore, which enables them to inhibit HMGR via competitive binding to the same binding pocket as the native substrate HMG-CoA.

Statin usage is associated with many secondary or pleiotropic effects [22]. A well-documented pleiotropic effect is statin muscle symptoms (SAMs) [23], which are broadly classified as a myopathy. However, SAMs also encompass symptoms such as myalgias (muscle pain without raised creatine kinase), myositis (muscle pain with raised creatine kinase), and rhabdomyolysis (severe muscle symptoms with creatine kinase more than 10-times normal) [24]. The frequency of SAMs is variable between study types. In randomized control trials, these symptoms occur in 1.5–3% of participants [24], when compared to observational studies occurring at a rate of ~10–33% [25]. A conservative estimate suggests that >1.5 million statin patients will experience SAMs annually [24]. The inconsistency in these rates has many potential explanations, including broad definitions of myopathy, exclusion of groups (i.e., previously statin-intolerant patients), and the nocebo effect [26,27]. The cause of statin-induced myopathy remains unknown, yet there is an expanding body of evidence for the relationship between lipophilicity and pleiotropy of a given statin [28,29,30]. This is based on the hypothesis that describes the biological effects of statins as being dependent on where they reside within cell membranes [28,29,30]. There is emerging, related evidence that describes the presence of possible statin transporters on the surface of skeletal muscle, which would allow for the extrahepatic accumulation of statins in skeletal myocytes [31]. Thus, current evidence is suggestive that the lipophilicity of statins, and therefore, how they associate with phospholipid membranes, is an important consideration when elucidating the cause of statin-induced myopathy.

Despite the harmful pleiotropic effects associated with statin use, many beneficial secondary effects are known. Statins are prescribed irrespective of serum cholesterol as secondary prevention for cardiovascular disease (CVD), including myocardial infarction, ischaemic stroke, atherosclerosis, and peripheral arterial disease [32,33,34]. Statins have also been explored in the treatment of non-cardiovascular diseases, eliciting beneficial effects in cancer, dementia and Alzheimer disease, bone repair, and non-alcoholic fatty liver disease [15,35,36,37,38,39,40]. However, the efficacy of statins in preventing and treating non-cardiovascular disease lacks convincing evidence to date. As such, this review will focus on statin–membrane interactions, as they pertain to the hypothesis of statin–membrane interactions causing SAMs.

In this review, we present a concise perspective on statin serum cholesterol reduction and reported statin–membrane interactions, offering mechanistic insight into statin pleiotropy, namely SAMs. The review starts with an introduction to cholesterol synthesis and the binding of statins to HMGR, highlighting the primary role of statins in treating hypercholesterolaemia. This is followed by details of the structure of the two main classes of statins and how this relates to the binding of statins to HMGR and their lipophilicity. The main part of this review consolidates the literature on statin–membrane interactions, particularly, where and how they reside within model phospholipid membranes. The combined knowledge from these studies highlights a possible relationship between lipophilicity, statin–membrane interactions, and statin myotoxicity. Related to this, we discuss how statins are metabolised and transported through membranes. Statins traffic through membranes by passive diffusion or via membrane transporters, whereby modulation of the membrane transporters has been speculated as a possible mechanism of statin myopathy. Finally, we briefly summarise the beneficial cholesterol-independent effects reported from statin treatment (refer to [41] for a review of this topic); however, discussion of novel drug delivery systems [42,43] to avoid or reduce adverse pleiotropic effects is beyond the scope of this review.

## 2. The Role of HMG-CoA Reductase in the Synthesis of Cholesterol

For over a century, cholesterol has been of great interest in science and medicine, from the initial discovery of *cholesterine* more than 200 years ago [44], its implication in atheromatous plaques in Virchow’s Triad [45,46], to the modern use of statins. In addition, adoption of the “lipid hypothesis” of atherogenesis was pivotal in the current understanding of how cholesterol and hypercholesterolaemia relate to coronary artery disease and myocardial infarction [47,48,49,50,51,52,53,54]. This began concurrently during the 1950s’ discovery of the clinically relevant relationship between cholesterol and atherogenesis. The importance of sterols is also highlighted by the fact that over the past 100 years, four Nobel Prizes were associated with sterol compounds and in particular, cholesterol synthesis and metabolism. They include: the 1927 and 1928 Nobel Prizes in Chemistry awarded to Heinrich Otto Wieland and Adolf Windaus, respectively, for their contributions to understanding the structure of bile acids and sterols, including the tetracyclic molecular formula of cholesterol [55,56,57]; the 1964 Nobel Prize in Physiology or Medicine awarded to Konrad Bloch and Feodor Lynen for uncovering the complex biosynthetic pathway involving thirty enzymatic reactions in the production of cholesterol [58,59]; and the 1985 Nobel Prize in Physiology or Medicine awarded to Michael S. Brown and Joseph L. Goldstein for their “discoveries concerning the regulation of cholesterol metabolism”, which formed the basis for the development of statins.

### 2.1. Cholesterol Biosynthesis

**Figure 1:**
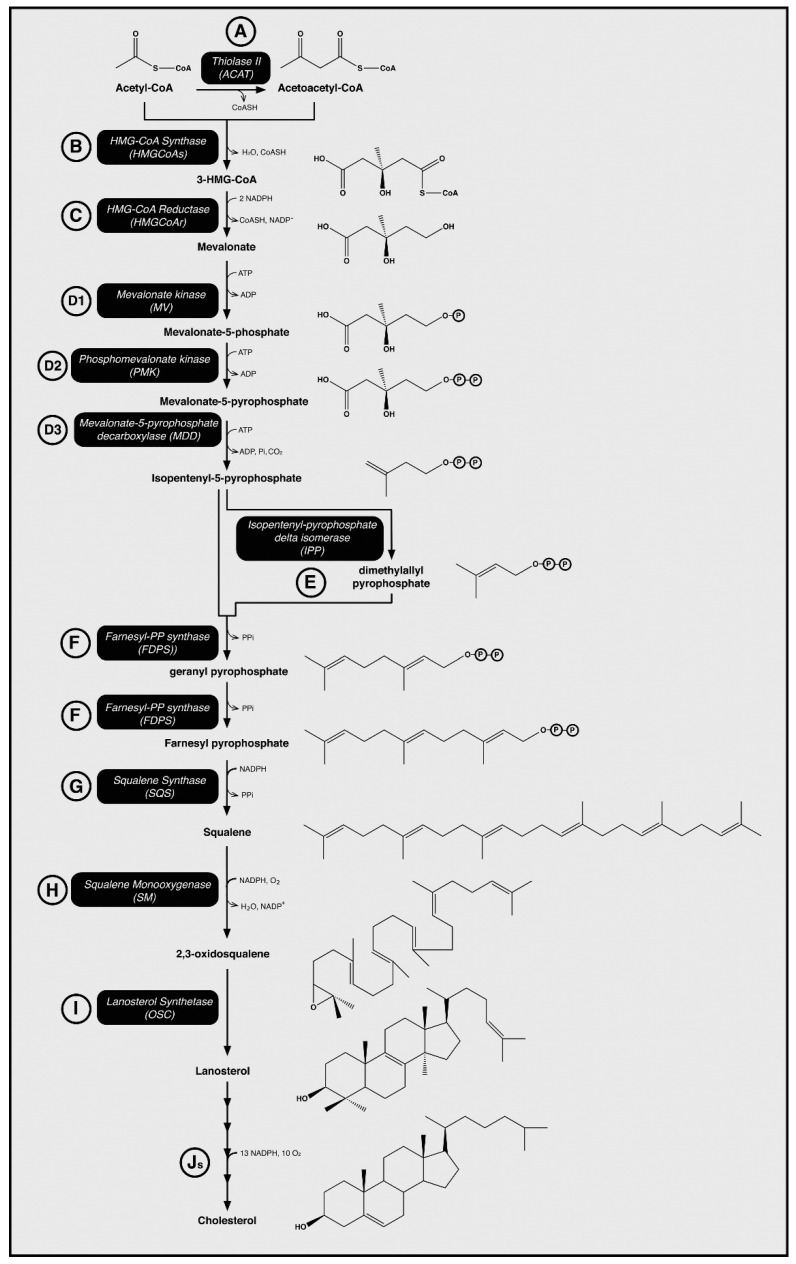
The cholesterol biosynthesis pathway and enzymes involved. Enzymes are labelled with letters in alphabetical order and referred to within the text; “s” indicates more than one enzyme in the step. Reprinted with permission from Cerqueira et al., Cholesterol Biosynthesis: A Mechanistic Overview, Biochemistry 55 (2016) 5483–5506 [16]. Copyright (2016) American Chemical Society.

A preliminary step in developing an intervention for hypercholesterolaemia is to understand the synthesis of cholesterol in mammalian cells. The complex biosynthetic pathway involves thirty enzymatic reactions in the production of cholesterol [58,59], which is outlined in Figure 1. The initial step of this pathway is the condensation of two acetyl-CoA molecules by the enzyme thiolase to form acetoacetyl-CoA (Step A in Figure 1). Then follows a condensation reaction between acetoacetyl-CoA and another acetyl-CoA molecule to produce HMG-CoA. This condensation is catalysed by 3-hydroxy-3-methyl-glutaryl-coenzyme A synthase (HMGS) (Step B in Figure 1). Next is the committed and rate-limiting step of cholesterol biosynthesis—the NADPH-dependent reduction of HMG-CoA into the six-carbon molecule mevalonate by HMGR (Step C in Figure 1) [16]. The next step is the ATP-dependent phosphorylation of mevalonate via mevalonate kinase (MV) to produce 5-phosphomevalonate (Step D1 in Figure 1) [60]. From here, the C_5_, isoprenoid isopentenyl pyrophosphate (IPP) is formed by means of phosphorylation and carboxylation, which is then isomerized yielding dimethylallyl pyrophosphate (DMAPP) (Step E in Figure 1) [61,62]. This isoprene molecule follows a series of condensation reactions to form the C_30_, linear compound, squalene (Step G in Figure 1). Squalene is the initial steroid precursor in metabolism, and its linear conformation is cyclised by oxidosqualene cyclase, yielding lanosterol, a quad-ring molecule. Following a 19-step conversion of lanosterol, a single molecule of cholesterol [63] is produced. Most pharmacological interventions to lower cholesterol levels in the blood, including statins, competitively inhibit HMGR and thus, prevent the formation of mevalonate and the subsequent formation of cholesterol.

### 2.2. HMG-CoA Reductase

HMGR is an enzyme that is highly conserved within all three domains of life (eukaryotes, prokaryotes, and archaea). The enzyme is not only pivotal in the formation of sterols, but also other biological molecules including derivatives of isoprenoids, like ubiquinone (CoQ10) and prenylated proteins [64]. The physiological importance of HMGR is evident as HMGR-knock-out mice exhibit embryonic lethality [65]. Despite its conserved nature, analyses revealed a discrepancy within the enzyme, resulting in a class distinction within the HMGR enzyme; that is, Class I and Class II. Class I consists of the NADPH-dependent catalytic synthesis of mevalonate in eukaryotes/mammals (Enzyme Commission number 1.1.1.34). Class II includes the NADH-dependent catalytic synthesis in prokaryotes (Enzyme Commission number 1.1.1.88) [66,67].

**Figure 2:**
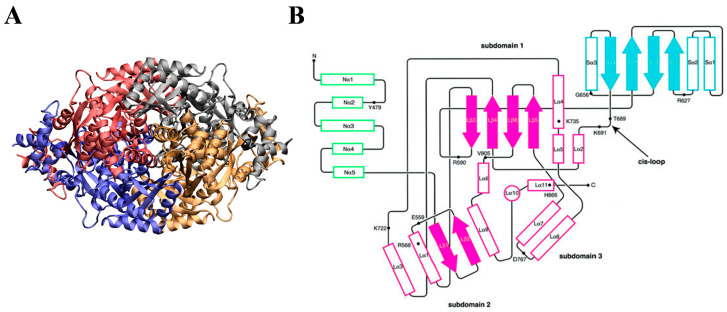
Structure and topology of HMG-CoA Reductase. (**A**) Crystal structure of human HMG-CoA Reductase with each monomer of the homo-tetrameric protein shown in a different colour (red, blue, grey, orange). Images prepared with Visual Molecular Dynamics (VMD) [71] and the Protein Data Bank (PDB) structure 1HWK.pdb. (**B**) Topological structure of a human HMG-CoA Reductase monomer, with each monomer domain defined by a different colour. N-domain indicated in green; L-domain (and its subdomains) indicated in magenta; S-domain indicated in cyan. Outlined boxes represent helices, and solid arrows represent strands. N- and C-termini are indicated. Figure taken from Istvan et al., EMBO, 2000 [68], with permission from John Wiley and Sons 2020.

Crystal structures of eukaryotic HMGRs revealed that the protein functions as a tetramer (four monomers), arranged to produce a dimeric active site at the interface of two monomers (Figure 2A). As illustrated in Figure 2B, each monomer consists of an N-terminal domain (N-domain, green), a large-domain (L-domain, magenta), and a small-domain (S-domain, cyan), linked by a *cis*-loop (amino-acid residues 682–694), which is necessary to form the catalytic binding site [68]. The N-domain forms a hydrophobic, integral membrane protein that anchors it to the endoplasmic-reticulum (ER) membrane. The L-domain is responsible for substrate binding (i.e., HMGR) and the S-domain binds NADPH [66,69].

Except for Class I-specific archaea, the structure of Class II HMGRs are similar to those within Class I but lack an N-domain. Moreover, because Class II HMGRs lack this membrane-binding domain, they dissolve within the cytosol [16]. Using sequence analysis, it was found that the catalytic domain is well conserved between the two classes, with approximately 60% sequence homology [16,68]. The N-terminus is poorly conserved between eukaryotes, with <25% homology between human and fungal HMGR [70].

## 3. The Structure of Statins

**Figure 3:**
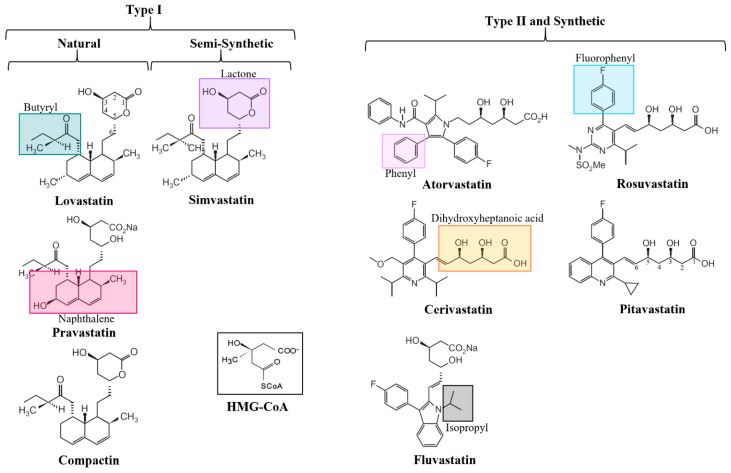
Classification of statin drugs. Statins are classified based on their chemical structure (Type I and II), or synthesis (Natural, Semi-Synthetic, and Synthetic). HMG-CoA, the natural substrate of HMGR, does not fall into any category. The HMG-CoA-like pharmacophore lactone is isolated in purple. The butyryl group of Type I statins is isolated in green. The naphthalene ring of Type I statins is isolated in magenta. The phenyl group of Type II statins is isolated in pink. The fluorophenyl group of Type II statins is isolated in blue. The dihydroxyheptanoic acid pharmacophore of Type II statins is isolated in orange. The isopropyl group of Type II statins is isolated in grey.

Statins are divided into classes based either on their synthesis or their structure. In the case of synthesis, three classes exist: natural, semisynthetic, or synthetic (Figure 3) [72]. Natural statins are produced by fermentation of fungi and include lovastatin, compactin, and pravastatin. Semi-synthetic statins, including simvastatin, are synthesised by the alkylation of lovastatin, replacing the 2-methylbutyrate moiety at the C-8 position of the naphthalene ring, with 2,2-dimethylbutyrate [73,74,75]. Synthetic statins are chemically synthesised, and only share the common dihydroxyheptanoic acid pharmacophore (HMG-CoA-like) moiety, which mimics the HMG-CoA substrate in HMGR binding. Synthetic statins include atorvastatin, cerivastatin, rosuvastatin, fluvastatin, and pitavastatin. All statin forms can be reversibly metabolised via lactonization (discussed in Section 7.1 below).

Structural classification is based on the type of ring structure found in the statin (Figure 3). Type I statins, including lovastatin, compactin, and simvastatin, contain a naphthalene ring. Type II statins all contain a fluorophenyl group and a series of hydrophobic ring structures that are covalently attached to an HMG-CoA-like moiety; specifically, pyrrole in atorvastatin, pyridine in cerivastatin, pyrimidine in rosuvastatin, indole in fluvastatin, and quinoline in pitavastatin. These hydrophobic ring structures mimic the HMG-CoA substrate and are important in binding to the HMGR. Side groups branching from the rings modulate the solubility/hydrophilicity of the drug and influence its pharmacokinetics—particularly, the butyryl moiety in Type I, and fluorophenyl and isopropyl in Type II [15,73].

## 4. The Interaction of Statins with HMGR

### 4.1. Thermodynamics of Statin-HMGR Interactions

Statins reversibly bind to HMGR but bind at a higher affinity when compared to HMG-CoA. Here, HMG-CoA binds with a K_m_ = 4 μM, compared to statins which exhibit a K_i_ (enzyme inhibitor binding constant) in the nanomolar range [73,76,77,78]. The K_i_ of different statins is shown in Table 1. There is a difference between the K_i_ of Type I and II statins, in which Type II are generally more potent than Type I, except for simvastatin. This difference pertains to the physical structure of statins, with Type II statins having larger hydrophobic domains, which account for more inhibitory binding interactions with HMGR [73].

Thermodynamic parameters of statin and HMGR binding correlate with their structure (Table 1). In particular, the hydrophobic regions influence their thermodynamic behaviour significantly. In this, rosuvastatin has the lowest free energy (ΔG), reflective of its strong binding affinity to HMGR when compared to other statins. The proportion by which enthalpy (ΔH) and/or entropy (−TΔS) contributes to statin binding reflects the diversity of interaction within the statin drug class. Enthalpy describes the attractive interaction between ligand (statin) and enzyme, whereas entropy communicates the repulsion of the HMGR enzyme from the solvent (water), and relates to changes in conformational degrees of freedom in the enzyme, plausibly relating to the flexibility of the HMGR C-terminal residue [76,77]. At 25 °C, rosuvastatin binding is predominantly enthalpically driven, accounting for 76% of its binding thermodynamic characteristics, compared to nearly all other statins, which all have an enthalpic binding contribution of < ~40%. Albeit, at physiological temperatures (37 °C), binding enthalpy (ΔH) increases and rosuvastatin binding is solely dominated by enthalpy. Studies of thermodynamic properties of stain-HMGR binding reported that protonation and deprotonation have negligible effect on the enthalpy of statin binding. Therefore, a proportional correlation between enthalpy and binding affinity exists, aligning with other ligand–enzyme interactions [79,80]. However, this conclusion was only produced on a small dataset [77].

The burial of polar and non-polar regions, namely from the hydrophobic ring structures, differs within the drug class due to variation in the number and type of polar atoms involved. This is expressed by the solvent-accessible surface area (SASA) [81,82]. The polar SASAs of several Type I statins are rosuvastatin (341 Å^2^), atorvastatin (323 Å^2^), cerivastatin (304 Å^2^), and fluvastatin (291 Å^2^) [77]. The increased burial of polar regions between these statins relates to the statins’ ability and availability to form H-bonds with HMGR. Conversely, the burial of the non-polar region surface area of the statins relates to the number of hydrophobic rings attached to the dihydroxyheptanoic acid pharmacophore. As such, atorvastatin (692 Å^2^) has the highest non-polar SASA, followed by cerivastatin (605 Å^2^), fluvastatin (593 Å^2^), and rosuvastatin (512 Å^2^) (Table 1). A large contribution to statins conformational entropy to HMGR is via bond rotation. Fluvastatin binding is almost solely driven by entropy because of its limited number of rotatable bonds. It has a negligible enthalpic contribution to its ΔG, which is associated with a large desolvation cost to enthalpy (up to 20.92 kJ/mol) from its indole ring [77,83]. Evidence suggests that statin structural constituents account for binding entropy. Therefore, the presence of more oxygen moieties, especially in the case of atorvastatin and rosuvastatin, allows for higher H-bonding capacity, and more expansive ring-structures produce more non-polar binding interactions.

**Table 1:** Various measure of statin lipophilicity, efficacy, and binding dynamics. Ki = enzyme inhibitor binding constant, loP and logD. ΔG = Free-energy (kcal/mol) of HMG-CoA binding at 25 °C. ΔH = Enthalpy (kcal/mol) of HMG-CoA binding at 25 °C. −TΔS = Entropic contribution (kcal/mol) of HMG-CoA binding at 25 °C. SASA_polar_ and SASA_non-polar_ (Å^2^) are the solvent-accessible surface area for the entropy estimation. LoP and LogD are partition coefficients (water-octanol) at pH 7. Efficacy of statins as measured by LDL % reduction at 40 mg dosage.

	Type II					Type I		
	Atorvastatin	Cerivastatin	Rosuvastatin	Fluvastatin	Pitavastatin	Simvastatin	Pravastatin	Lovastatin
***K*_i_ (nM)** [76]	8.0 (6.0–12.0)	10.0 (7.0–15.0)	3.5 (2.5–5.0)	28.0 (18.0–42.0)	−	11.0 (7.0–17.0)	44.0 (29.0–66.0)	−
**ΔG (kcal/mol)** [77]	−10.9 ± 0.8	−11.4 ± 0.4	−12.3 ± 0.7	−9.0 ± 0.4	−	−	−9.7 ± 0.4	−
**ΔH (kcal/mol)** [77]	−4.3 ± 0.1	−3.3 ± 0.2	−9.3 ± 0.1	~0	−	−	−2.5 ± 0.1	−
**−TΔS (kcal/mol)** [77]	−6.6 ± 0.6	−8.1 ± 0.4	−3.0 ± 0.7	~−9.0	−	−	−7.2 ± 0.4	
**SASA_polar_ (Å^2^)** [77]	323	304	341	291	−	−	−	−
**SASA_non-polar_ (Å^2^)** [77]	692	605	512	593	−	−	−	−
**log P (pH 7)** [84,85]	1.61	2.05	0.13	1.67	−	2.06	−0.23	1.7
**log D (pH 7)** [86]	1.53	2.32	−	1.75	1.5	4.4	−0.47	3.91
**LDL % Reduction at 40 mg dosage** [87]	48	−	60	27	−	41	34	34

### 4.2. Molecular Interactions in the Statin-HMGR Complex

**Figure 4:**
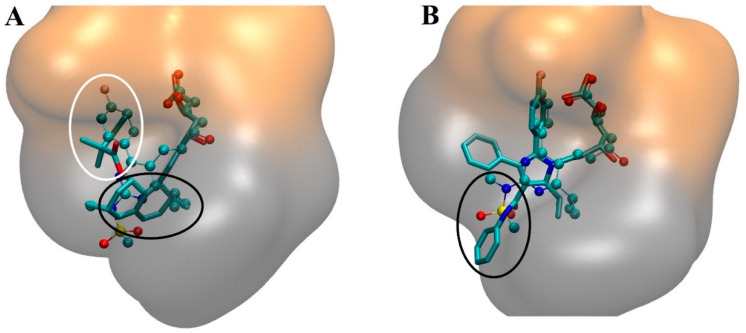
Binding of statins to HMGR (**A**) Overlay of Type I, simvastatin (stick) and Type II, rosuvastatin (ball and stick) in the HMGR binding site at the interface between two HMGR monomers (orange and grey) represented as a surface model. The binding site was defined by any HMGR residues within 4Å of any atom of a statin molecule. Circled in black is the common binding region of the naphthalene ring (Type I) and isopropyl (Type II). Circled in white is the common binding region of the butyryl group (Type I) and fluorophenyl group (Type II). (**B**) Overlay of atorvastatin (stick) and rosuvastatin (ball and stick) at the HMGR binding site between two HMGR monomers (orange and grey) represented in a bubble surface model. Circled in black are the common binding region of a carbonyl group (atorvastatin) and a sulphonamide group (rosuvastatin). Both images were prepared with VMD [71] and the PDB structures 1HW9.pdb and 1HWL.pdb.

Statins bind to the L-domain of the HMGR protein, indicated in magenta in Figure 2B. Specifically, the HMG-CoA-like pharmacophore occupies the *cis-loop* with the hydrophobic ring structures also residing within the HMGR L-domain, sterically impeding HMG-CoA substrate binding. There are several polar interactions made with the HMG-CoA-like pharmacophore and the amino acid residues within the HMGR *cis*-loop. In the case of type II statins, the C5-hydroxyl group of the statin replaces the thioester oxygen of HMG-CoA. The bulky hydrophobic ring structures of the statins result in multiple polar interactions with HMGR, and the diversity of structure within the drug class causes different conformations of statins to maximise contact with the protein [73,88]. Moreover, the innate flexibility of the C-terminal residues of HMGR is utilised in the binding of the hydrophobic rings of statins, allowing them to reside in a groove that would otherwise house the pantothenic acid moiety of CoA [73,89,90]. Despite the diversity of hydrophobic groups, the structural differences between Types I and II functionally serve the same purpose; the naphthalene ring of Type I statins occupying the same site as the isopropyl of Type II, and the butyryl moiety of Type I statins replaces the fluorophenyl group in Type II statins (Figure 4A). Synthetic, or Type II, statins are known to have more binding interactions with HMGR due to a more expansive hydrophobic structure. Rosuvastatin and atorvastatin have more hydrogen-bonding interactions with HMGR, in which they bind to the same amino acid residue using their sulphonamide or carbonyl group, respectively (Figure 4B) [73,88]. It should also be noted that statins do not impede on the NADPH binding pocket, formed by the S-domain in HMGR [91].

In addition to hydrophobic/non-polar interactions, electrostatic or polar interactions are important to understand the binding of statins to HMGR [81,82]. Surface electrostatic models, based on quantum mechanical calculations of statins [92], showed high negative charge density in the HMG-CoA-like moiety, resultant from the two oxygen atoms at C-1 and hydroxyl groups at C-3 and C-5 (Figure 3). The side chains of Type I statins are capable of polar interaction by virtue of their butyryl group, and some polar interactions are possible in Type II statins due to a π-electron cloud of phenyl side chains. The bond strength (~1 kcal/mol) of these π-electron cloud interactions are comparable to dipole-dipole interactions (1 kcal/mol) or weak hydrogen bonds (1–10 kcal/mol) [92]. Rosuvastatin and atorvastatin have strong electrostatic charges on a methyl group of the isopropyl moiety, resulting from electrostatic interaction with amine groups on the adjacent pyrimidine or pyrrole rings, respectively. The effect of this is suspected to be hyperconjugation between the isopropyl group and the leucine (Leu562) amino acid residue of HMGR [93]. As such, the polarity of statin binding makes them highly sensitive to steric interaction and conformational changes.

Conformational changes in statin binding to HMGR occur within the dihydroxyheptanoic acid inhibitor moiety, with changes of interest being the C-1 carboxylate and C-3 hydroxyl groups. Because of the polarity of these oxygen atoms, a strong electrostatic field exists between these two groups, with an energy difference between the C-1 moieties and C-3-hydroxyl of ~288 kcal/mol [92]. This energy differential allows for stabilisation of the C-3 hydroxyl group in its lowest energy form; that is, the C-3 hydroxyl at 60° from the C-1 carboxylate. Moreover, electrostatics of the statin pharmacophore is imperative to its binding efficacy as HMGR binding is stereoselective, where the C-3 and C-5 hydroxyl groups on the statin pharmacophore are active and stable in a 3R,5R enantiomer compared to the inactive 3S,5R enantiomer. The active configuration on HMGR binding allows for solvation and desolvation changes, which facilitates appropriate enzyme-ligand binding [92].

## 5. Interaction of Statins with Membranes

### 5.1. Lipophilicity of Statins

Statin lipophilicity is important due to its relationship with hepatoselectivity (discussed in Section 6 below) and its implication in statin association with extra-hepatic tissue, like skeletal muscle, which is speculated to cause SAMs. The hydrophilicity or lipophilicity of statins is related to its structural constituents [15,73]. The hydrophilicity of statins originates from the common HMG-CoA-like pharmacophore, and other highly polar substituents like carboxylate and hydroxyl groups, or from sulphonamide moieties in the case of rosuvastatin. Alternatively, lipophilicity results from their hydrocarbon ring structure and non-polar substituents like isopropyl, phenyl, and fluorophenyl groups. Although the H-bonds that form between the statin and lipids or proteins are much stronger than the van der Waals interactions of the hydrophobic regions, the multitude of the non-polar interactions approximates that of polar bonding, and statins are thus considered amphiphilic molecules [92]. The lipophilicity of these drugs is therefore critical to the pharmacokinetics and pharmacodynamics and are thought to be involved in the cholesterol-independent or pleiotropic effects of statins [15,73,94,95,96].

The most widely used method of communicating statin lipophilicity is through the logP partition coefficient (typically water and n-octanol) for unionised drugs, or logD for ionised drugs [84,97,98]. P is the partition coefficient and describes the concentration ratio of the distribution of a molecule between a polar (i.e., water) and non-polar (i.e., n-octanol) solvent [99]. This has been a highly useful way of conveying the affinity of statins to phospholipid bilayers (Table 1), as the hydroxyl group of n-octanol simulates the H-bonding of statins with ester and carboxylate groups of phospholipids. However, several impediments in partitioning experiments have been identified; namely, changes in solubility can result in an error (in equilibrium, n-octanol contains 2.8 M water in partitioning experiments meaning polar solutes can solvate). Therefore, the use of partition coefficients as a proxy for phospholipid bilayer partitioning may be misleading [92]. It has since been shown via bimolecular simulations that n-octane is superior to n-octanol in determining logP, despite other solvents being utilised in the past (i.e., heptane) [100]. Because n-octanol and water elicited analogous bulk solvent energies, they have similar H-bond forming capacities, so by substituting n-octane, it allows for identical carbon chain length, with the benefit of negligible/no H-bonding capacity. Despite this finding, n-octanol in partition coefficient calculations remains the standard [92,101,102,103].

### 5.2. Statin–Membrane Interactions

Evidence suggests that a relationship exists between statin lipophilicity and pleiotropy, which supports the hypothesis that the effects of statins are dependent on where it resides within biological membranes [28,29,30]. It is known that the inclusion of amphiphiles into lipid bilayers impacts lipid bilayer parameters like permeability and thickness [104,105]. Moreover, amphiphilic drugs are soluble in aqueous biological fluids and can diffuse through the body, and partition into membranes [106,107]. Statins also bind onto phospholipids on the surface of LDL [108]. Additionally, statins were observed to alter lipid bilayer properties by increasing membrane elasticity, with fluvastatin being the most effective membrane disruptor, and rosuvastatin the least [109]. Moreover, a study using atomic force microscopy (AFM) described that statins increase the dispersity of membrane elasticity, suggesting statins are heterogeneously miscible in lipids [110]. The unique lipophilicity of each statin will naturally impact how deep into the membrane they can penetrate and, per the current hypothesis, indicate how strong the membrane altering effects are. There is limited information within the literature about statins’ effects on biological membranes. Most studies discussed in this review utilise phosphatidylcholine (PC) lipids as they are the predominate phospholipids in mammalian cells. PC lipids contribute the most of any lipid, occupying ~33% of plasma membranes, and >50% of the ER membrane, where HMGR acts [111].

**Figure 5:**
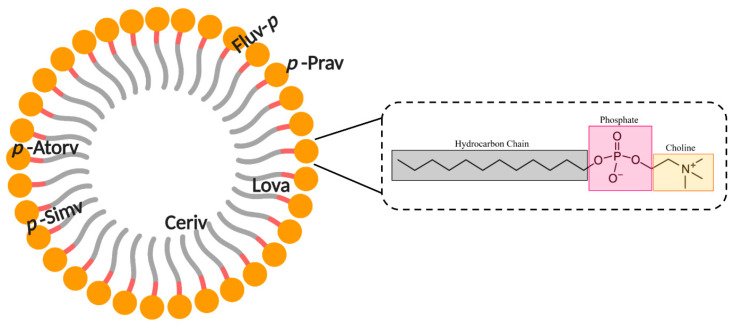
Schematic representation of dodecyl phosphocholine (DPC) micelle with approximate binding locations of statins (atorvastatin, cerivastatin, fluvastatin, lovastatin, pravastatin, and simvastatin). The orientation of the statin within the micelle is presented, whereby “p” is the statin pharmacophore. Cerivastatin does not have a specified orientation due to its location in the DPC micelle core. Lovastatin’s orientation within DPC micelles was unquantifiable. Magnified is DPC with labelled chemical moieties. Adapted from Galiullina et al., 2017 [114]; Galiullina et al., 2018 [115]; Shurshalova et al., 2020 [112]. Created with BioRender.com.

The introduction of lovastatin in dodecyl phosphocholine (DPC) micelles is a simple model to mimic statin and biological membrane interactions. Using nuclear magnetic resonance (NMR), it was observed that lovastatin does not have a specific orientation within the lipid part of the micelle, albeit is fully embedded in the membrane [112]. On average, lovastatin is located within the hydrophobic, hydrocarbon tails in the non-polar inner part of DPC, but it is not possible to identify a specific position, which indicates a dynamic positioning of lovastatin [28,113] in the micelle. Further, it was determined that lovastatin penetrates the DPC hydrophobic tails with its butyryl and lactone ring deeper than its naphthalene ring structures, between 14 and 15 Å from the micelle centre of mass (Figure 5) [112]. Similarly, in another study, it was observed that lovastatin, when bound to a planar, 1-palmitoyl-2-oleoyl-sn-glycero-3-phosphocholine (POPC) bilayer membrane, induced no effect on lipid packing, in spite of its dynamic orientation, and could not be accurately located, nor its orientation resolved [113].

Simvastatin was seen to be located in a similar space to lovastatin, but it exhibits a more defined location and orientation within the membrane [114,116]. It locates between the acyl chains of DPC, its naphthalene ring structure has hydrophobic interactions with these chains, and the inhibitor moiety has ionic interactions with the polar PC head groups [28,114]. Simvastatin is identical to lovastatin, with the exception of an additional methyl group on the second carbon of the butyryl chain. This suggests that even minor alterations in statin structure can have significant impacts on the membrane interactions of statins. Calorimetric investigations using simvastatin in 1,2-dipalmitoyl-sn-glycero-3-phosphocholine (DPPC) vesicles observed simvastatin binding within the DPPC acyl chain between C2 and C8 via changes in membrane cooperativity [117,118]. It was also observed that the carbonyl oxygen of the HMG-CoA-like moiety resides at the lipid interface, and is capable of H-bonding with the water at the water–lipid interface, or with adjacent glycerol groups in phospholipids [117]. Simvastatin has been shown to impact the acyl chain order and therefore, alter lipid packing in a depth-dependent and phase-specific way. This has been investigated in different and changing lipid phases, in which the addition of simvastatin saw a phase change from gel to liquid-crystalline and saw lateral phase separation of DPPC lipids. This indicates that simvastatin amplifies membrane elasticity and fluidity, alters membrane curvature in unilamellar vesicles, and increases lipid acyl chain disorder, creating fluctuations in the lipid state during phase transitions [117]. A similar investigation using fluorescence measurements, which utilised separate lipid compositions as mimetic phase changes, saw the most significant effect in DPPC membrane order, which represented the gel phase [119]. Although, in POPC membranes, the fluid phase saw negligible change in lipid order or polarity. Conversely, POPC with 40% cholesterol forms the liquid-ordered phase [111,120,121,122], which represents a middle-ground between the aforementioned phases; small changes in membrane parameters were observed [119].

**Figure 6:**
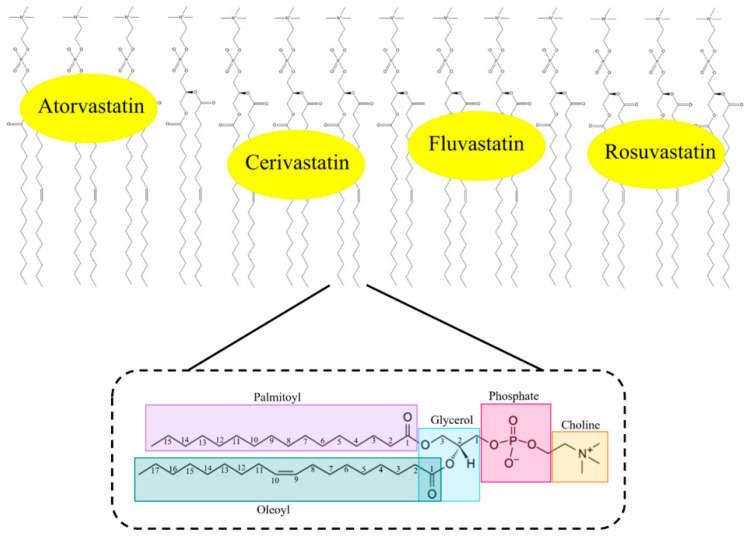
Schematic representation of the approximate binding locations of statins (atorvastatin, cerivastatin, fluvastatin, rosuvastatin) within POPC lipid bilayers. Magnified is a POPC lipid with labelled chemical moieties. Reprinted from Biochimica et Biophysica Acta (BBA)–Biomembranes, 1861, Galiullina et al., Interactions of statins with phospholipid bilayers studied by solid-state NMR spectroscopy, 10, [115] Copyright (2018), with permission from Elsevier.

Atorvastatin has been shown to maintain a linear and unfolded structure when complexed with DPC micelles, which parallels other statins [92]. Using NMR, it was shown that atorvastatin penetrates membranes leading with its hydrophobic ring structures, which embed themselves between the aliphatic tails of DPC. Here, the alanine group resides the deepest within the micelles. Conversely, the polar, dihydroxyheptanoic acid moiety remains at the interfacial region of the micelle, and therefore, does not completely permeate the membranes (Figure 5) [115]. As such, it behaves almost identically to simvastatin, in that it resides between the aliphatic tails, and in between the PC head groups and the micelle core, forming H-bonds at the lipid interface [117]. Within a more physiologically relevant POPC membrane model, the location of atorvastatin remains similar (Figure 6). The deepest aromatic ring is once again the alanine group, located in the upper portion of the acyl chains, with the other fluorophenyl and phenyl groups taking a shallower position in the glycerol region of the lipid tails (Figure 6). It is reported that the inhibitor moiety was not resolved in this case, albeit it was speculated to reside at the lipid interface as per the DPC micelle model [113,115]. This relatively shallow association with these membranes is attributed to the cation–π interactions between the statin rings and the neighbouring lipid structures—in this case, the phosphate and glycerol moieties [113].

Fluvastatin was calculated to be ~79% bound to DPC in a D_2_O suspension, which is lower compared to other statins (88% cerivastatin and 84% pravastatin) [114]. This may be in part due to the way that fluvastatin penetrates into the membrane. It does so by using its lipophilic ring structures to anchor in a shallow incorporation into DPC micelles, leaving the HMG-CoA-like pharmacophore as a non-penetrating feature (Figure 5). Electrostatic changes between the polar PC head groups, hydrophobic rings, and the micelle’s apolar core are said to play an important role in its membrane association, and its permeability through jejunum cell walls [114,123,124]. This result from studies with DPC micelles is mirrored in solid-state NMR using POPC membrane models (Figure 6). Results suggest that the hydrophobic regions, particularly the indole group, resides the deepest at approximately the C-2 level of the palmitoyl acyl chain, and the HMG-CoA-like pharmacophore facing towards to head groups [113].

Pravastatin only shows surface interactions with DPC micelles and does not permeate the mimetic membrane (Figure 5). The binding is governed by weak electrostatic interaction between the inhibitor and butyryl moieties and the PC headgroups. This can be explained in part by the weak lipophilicity of pravastatin. It was also shown that pravastatin has a similar self-diffusion coefficient as DPC micelles, indicating weak associations between pravastatin and DPC [114]. This would aid in explaining the necessary active transport systems in place for pravastatin transport into and out of hepatocytes [86,125,126,127]. Despite its shallow binding to lipids, compared to most other statins, pravastatin shows a strong effect on chain order at the C-12 acyl chain level in POPC membrane models. Only pitavastatin showed a stronger effect. [113].

Rosuvastatin is a hydrophilic statin and as such, shows the lowest binding to POPC membranes (~8%) when compared to nearly all other statins. It was similarly reported that the hydrophilic statins saw the lowest binding to POPC membranes when compared to their lipophilic counterparts (proportion bound: ~0%/non-detectable pravastatin, 15% fluvastatin, 29% atorvastatin, 36% cerivastatin, 70% lovastatin). The incorporation of rosuvastatin into the membrane was only able to alter the PC lipid head group orientation and mobility, as determined via chemical shift anisotropy [113]. The ring structures of rosuvastatin were observed to reside between C-3 of the glycerol group and C-3 of the palmitoyl acyl chain of POPC in solid-state NMR studies. It is described to be mobile within the membrane, and its orientation is unknown, albeit its approximate location is close to C-2 and C-3 of the palmitoyl acyl chain (Figure 6) [113].

For cerivastatin, NMR spectroscopic studies showed that it inserts into DPC micelles, such that it penetrates so deeply that it resides completely within the micelles’ hydrophobic core, indicating it has very strong hydrophobic interactions (Figure 5) [114]. This parallels results using POPC membrane models, as cerivastatin saw the deepest penetration within the drug class; however, this was not to the extreme depth observed within DPC micelles. Here, its aromatic rings were located slightly deeper than the C-3 level of the palmitoyl acyl chain, yet its orientation within the space remains unknown (Figure 6) [113]. The lipophilicity of cerivastatin is reflective of its increased rates of myotoxicity and rhabdomyolysis (pathological muscle breakdown). This is in contrast to pravastatin and fluvastatin, which offer little-to-no membrane penetration and saw some of the lowest rates of rhabdomyolysis within the drug class [28,128,129]. Cerivastatin’s deep penetration into membranes may cause interference with integral membrane proteins like *multidrug resistant-associated protein*s (MRPs) within skeletal myocytes [114].

## 6. Lipophilicity and Hepatoselectivity

Lipophilicity of statins is of particular clinical importance for two main reasons: (a) hydrophilic statins are excreted largely unchanged, while lipophilic statins undergo oxidative biotransformation by the CYP450 family and are thus, susceptible to drug-drug interactions (DDI) [130,131,132,133]; (b) lipophilic statins have the propensity to passively and non-selectively passage through the membranes of non-hepatic tissue [92].

Fibrates like *gemfibrozil* and immunosuppressants like *cyclosporin* are inhibitors of the statin transporter *organic anion transporting polypeptide* 1B1 (OATP1B1) and therefore, pose a high risk of adverse pleiotropic effects (detailed in Section 7.2) [131,132,133]. This explains the contraindication for co-administration, lethality, and eventual removal of cerivastatin from the market in 2001. Cerivastatin resulted in rhabdomyolysis and acute kidney failure from circulating myoglobin at a rate 10-times greater than all other statins [134]. Hepatoselectivity, which is the preference of a drug to associate with hepatocytes as its primary target, is thought to reduce the risk of pleiotropic effects like statin-associated muscle symptoms (SAMs) and rhabdomyolysis. Avoiding statin–muscle interaction is vital, as skeletal myocytes have a cholesterol inhibitory sensitivity 40-times greater than hepatocytes [135]. Hepatoselectivity is related to each statin’s lipophilicity, where lipophilicity reflects the molecules’ ability to passively travel through cellular membranes, particularly in tissue that is exogenous to the liver [28,29]. As such, lipophilic statins will passively and non-selectively diffuse into non-hepatic tissue, particularly skeletal muscle and across the blood–brain barrier. Conversely, hydrophilic statins, like rosuvastatin and pravastatin, traffic into cells using OATP active uptake transporters. Statins’ efficacy is in part due to their lipophilicity and how quickly they can partition through membranes; which is associated with increased rates of drug metabolism, clearance and reductions in drug accumulation within the body, and necessary dosage adjustments [86]. Therefore, lipophilicity is important in statin bioavailability and bioactivity [15,136].

In designing novel statins with the aim of reducing non-specific and non-hepatic binding at the forefront of design, strong hepatoselectivity and low lipophilicity are crucial. Evidence suggests that amine residues, particularly cycloalkyl sulphonamide analogues, have the most potent hepatoselectivity and strong cholesterol-synthesis inhibition in vivo. Notably, an inverse proportionality was found between calculated logP (ClogP) values and the IC_50_ for myocyte binding (i.e., increase in lipophilicity creates less hepatoselectivity) [137]. This aligns with rosuvastatin, which is a hydrophilic statin, containing the most amine residues, and is the most potent statin currently available (Table 1) [87].

## 7. Statin Metabolism

### 7.1. Lactonization

**Figure 7:**
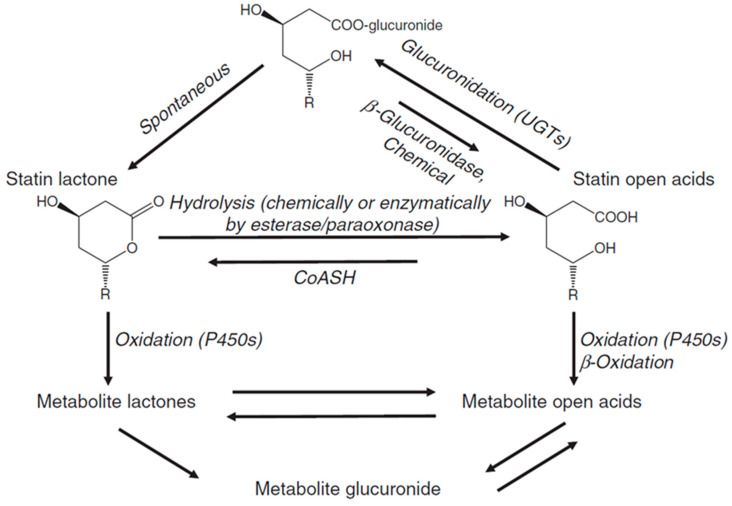
Lactonization or glucuronidation of statins. Conversion of statin acids into statin lactones is mediated by UDP-glucuronosyl transferase (UGT), or by the CoASH-dependent pathway. This reaction is reversible and occurs via hydrolysis of the lactone by esterases or paraoxonases. Statins have an oxidative interconversion pathway which involves the Cytochrom P450 family. Reprinted from Pharmacology and Therapeutics, 112, Shitara and Sugiyama, Pharmacokinetic and pharmacodynamic alterations of 3-hydroxy-3-methylglutaryl coenzyme A (HMG-CoA) reductase inhibitors: Drug–drug interactions and interindividual differences in transporter and metabolic enzyme functions, 35, [86] Copyright (2006), with permission from Elsevier.

Statins are administered orally in a lactone form (Type I statins), acid form, or an anionic-salt form. The lactone, or closed-ring, form of statins is the inactive form and is reversibly hydrolysed into the active dihydroxyheptanoic acid form via carboxylesterases in the liver, intestinal wall, and plasma. Approximately 10% of the administered dosage of active statins can be converted into its lactone form via lactonization or glucuronidation. This is mediated by UDP-glucuronosyl-transferase (UGT) (Figure 7) [138,139]. In human serum, the statin dihydroxyheptanoic acid form is stable, whereas the lactone form is unstable and is readily hydrolysed. For atorvastatin and fluvastatin, under slightly basic conditions, the dihydroxyheptanoic acid form is energetically favoured. However, under acidic conditions, as H^+^ catalyses lactonization, the lactone forms are produced, albeit they are not physiologically favoured [140]. Both the lactone and acid forms of statins have been detected post oral dosage within human systemic circulation for atorvastatin, cerivastatin, rosuvastatin, simvastatin, and lovastatin [141,142,143,144,145]. Acid forms can be excreted into the biliary system, or converted into the lactone forms, to be metabolised by isoforms of Cytochrome P450 (CYP450). In this, Cytochrome P450 3A4 (CY3A4) is responsible for atorvastatin, simvastatin, and lovastatin metabolism [146,147,148]. Cytochrome P450 2C9 (CYP2C9) metabolises fluvastatin and to a lesser degree, pitavastatin [149,150,151]. Rosuvastatin, pravastatin, and pitavastatin undergo negligible metabolic interaction with CYP450 [151,152,153]. Cerivastatin undergoes metabolism via two CYP450 isoforms—Cytochrome P450 2C8 (CYP2C8) and CYP3A4. Metabolism of cerivastatin is predominantly performed by CYP2C8, which is evident by the fact that CYP3A4-inhibitors like erythromycin and itraconazole do not hinder metabolism. Its metabolism is susceptible to gemfibrozil, a CYP2C8-inhibitor [154,155]. Studies have also been conducted on determining the effects of CYP2C9 inhibitors, such as Amiodarone, on the pharmacokinetics of statins [156,157]. For a more in-depth review of potential interactions, refer to [158].

### 7.2. Hepatic Influx of Statins

**Figure 8:**
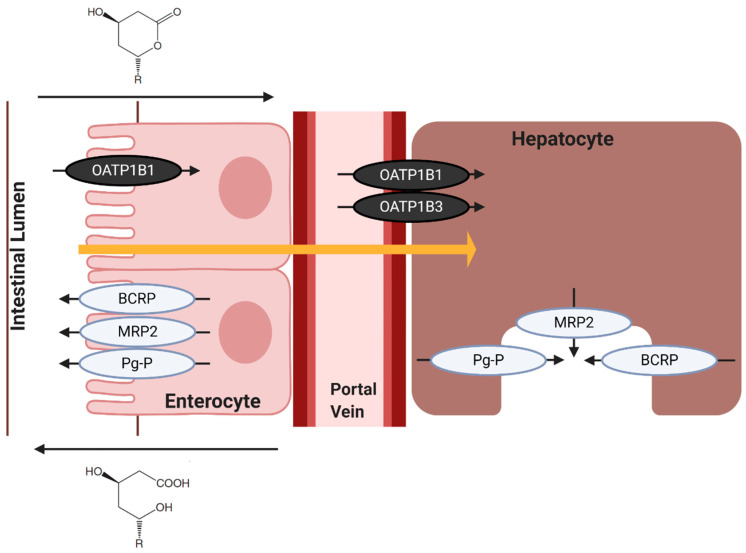
Schematic representation of statin influx to, and efflux from, hepatocytes. Here, black transporters indicate influx transporters from the intestinal lumen to enterocytes (OATP1B1), and from blood vessels into hepatocytes (OATP1B1 and OATP1B3). Transporters involved in the influx from enterocyte to blood vessel remain unknown. Efflux transporters are presented in grey (MRP2, BCRP, Pg-P). Here, they either transport statins from enterocytes into the intestinal lumen or into bile for excretion. Indicated at the top of the image is an arrow representing the preferential movement of statin lactones from apical-to-serous membranes, and at the bottom, an arrow indicating the preferential movement of statin acids from serous-to-apical membranes. The solid orange arrow represents the passive diffusion of lipophilic statins. Created with BioRender.com.

Uptake and transport into hepatocytes are critical to the metabolism and clearance of statins. Evidence suggests that there is a blend of passive diffusion and active transporters for entrance through hepatocellular membranes. In this, statin uptake is mediated by the Organic Anion Transporting Polypeptides (OATP), which is almost solely expressed on hepatocytes and is responsible for the uptake of bile acids, estrogens, and other endogenous compounds. Specifically, OATP1B1 transports and influxes statins into hepatocytes [159]. Statin metabolism and elimination are governed by the rate of hepatic uptake of these drugs [160]. Evidence suggests that nearly all statin variants are substrates of OATP1B1 (atorvastatin, cerivastatin, rosuvastatin, pravastatin, and pitavastatin) [86]. Pitavastatin has a dual uptake—with a predominant role in OATP1B1 and a minor contribution from OATP1B3. Alternatively, simvastatin and lovastatin are largely trafficked through membranes by passive diffusion, and its lipophilicity (as its ingested lactone form) allows for passive uptake into hepatocytes. However, the large size of statins means that OATP1B1 will have a minor contribution in simvastatin and lovastatin transport via facilitated diffusion (Figure 8) [161,162,163]. It should be noted that cerivastatin has a high, non-specific uptake by hepatocytes via passive diffusion, despite also being transported via OATP1B1, which is likely due to its lipophilicity [86].

Evidence comparing the active, dihydroxyheptanoic acid forms and the inactive, lactone forms of atorvastatin, simvastatin, lovastatin, and pravastatin in dog kidney cells saw an OATP1B1 (influx) uptake 3–7-times higher for statin lactones than dihydroxyheptanoic acid [164]. This is supported by results using human colorectal adenocarcinoma cell lines, Caco-2, monolayers in which Type II statin acids showed higher serous-to-apical membrane (traffic towards the lumen) transport rate when compared apical-to-serous membrane transport (traffic towards the blood vessel) (Figure 8) [165]. Similarly, a poor correlation of the transport of statin dihydroxyheptanoic acid via OATP in rat hepatocytes indicates that active forms of statins are not transported via OATPs [166]. Thus, it can be deduced that the uptake of statins by OATPs occurs in the inactive lactone form. Inhibitors of the OATP1B1 transporter include fibrates, namely gemfibrozil, and cyclosporin, which would cause increased plasma and/or, extrahepatic concentrations of statins [130,131,132,133].

### 7.3. Hepatic Efflux of Statins

The total body clearance or overall elimination of statins is governed by metabolic processes in the liver, kidney, and intestines. It has been shown that the hepatic uptake of statins is the rate-determining process in the overall elimination of statins. This was estimated using bile canalicular membrane vesicles [167,168], and a study of human and rat hepatocytes [169], in which the in vivo hepatic clearance and clinically defined intrinsic clearance for statins align. Different models of hepatic clearance of statins have also been produced—notably, one model in which the overall intrinsic clearance is influenced by metabolism and/or biliary excretion as well as membrane permeability. In this model, the heterogeneity/asymmetry in the ease of movement of statins to either influx into or efflux out of hepatocytes dictates the clearance of the drug. It describes the relationship between the membrane permeability in both statin influx and efflux, and how these changes influence the intrinsic clearance via metabolism or biliary excretion, and how these alter the overall hepatic clearance of unbound statins [86]. Most notably, if there is no asymmetry between the permeability of the hepatocyte membrane between influx and efflux of statin (which is representative of the passive diffusion of statins like simvastatin and lovastatin) [161], then the overall hepatic clearance of unbound statins is equal to the intrinsic clearance via metabolism or biliary excretion. When statins are able to rapidly penetrate through membranes, they remain relatively immune to DDI that would otherwise occur, namely, from interactions that impede transporters, like gemfibrozil [130,131,132,133].

Efflux transporters are crucial in transporting statins out of cells for elimination. It has been shown that one superfamily of plasma membrane proteins, the ATP-binding cassettes (ABC), plays a major role in the efflux of statins from hepatocytes. ABCs that are primarily involved in the removal of statins via active transport are *multidrug resistant-associated protein* (MRP2/ABCC2), *breast cancer resistance protein* (BCRP/ABCG2), and *P-glycoprotein* (Pg-P/MDR1/ABCB1) [160].

MRP2 is a highly active efflux transporter for statin and has been shown to be effective in transporting statins for biliary excretion in human MRP2 and in the rat counterpart, Mrp2 [168,170,171]. Its known statin substrates are quite expansive, including, atorvastatin, cerivastatin, rosuvastatin, pitavastatin, simvastatin, and pravastatin [126,127]. MRP2 is a unidirectional transporter on bile canalicular membranes for the excretion of anions. Its effects within the liver are pronounced as the expression of MRP2 in the duodenum had no correlation with systemic/serum statin concentration; however, increased expression of hepatic MRP2 mRNA resulted in a reduced systemic statin concentration, suggesting increased efflux and elimination [172,173].

BCRPs are widely expressed on normal cells, as well as cancer cells, and their involvement in biliary excretion of statins has been demonstrated using knockout mice in vivo and membrane vesicles in vitro [174,175]. They been linked to efflux of atorvastatin, rosuvastatin, pitavavstin, and pravastatin, as supported by evidence that ABCG2 polymorphism results in increased atorvastatin and rosuvastatin exposure [126,127,176].

Pg-P is an efflux transporter expressed on the apical membrane of liver canaliculi and cells of the gastrointestinal tract [177]. It has been shown that statins increase mRNA expression in LS180 cells (which have an epithelial morphology) and are similarly regulated in Caco-2 cells [178,179]. These transporters are present within many organ membranes, and similarly, transport many statin variants such as atorvastatin, cerivastatin, rosuvastatin, pitavastatin, simvastatin, pravastatin, and lovastatin [126,180]. A study of statins and efflux transporters Pg-P, MRP2, and Mrp2, showed a discrepancy between the lactone and dihydroxyheptanoic acid statin forms’ transport, in which the lactone forms’ IC_50_ is 10-times lower than the acid alternative [164].

## 8. Statin Transporters and Myopathy

The preferential uptake of statin lactones compared to dihydroxyheptanoic acid forms by membrane transporters has been implicated in myotoxicity, which corresponds with one of the most common secondary effects among statin users—myopathy [22]. High plasma levels of statin lactone have been observed in patients with statin-associated myopathy, where atorvastatin lactone, fluvastatin lactone, simvastatin lactone, and pravastatin lactone elicit a 14-, 26-, 37-, and 23-fold increase in potency to induce skeletal muscle cell cytotoxicity compared to its respective dihydroxyheptanoic acid form counterparts [181]. It is reported that statin lactone contribution to myotoxicity is due to the potent inhibition of (up to 84%) of mitochondrial complex III (CIII), the third integral membrane protein of mitochondria in the electron transport chain in ATP generation. Specifically, the Q_O_ binding site of CIII was observed to be the off-target site of statin lactones. Patients with statin-induced myopathy have a CIII activity reduction of ~18% [182]. Moreover, the presence of statin uptake and efflux transporters OATP2B1, MRP1, MRP4, and MRP5 has been described on the sarcolemma membrane of human skeletal myocytes, and as such, may be implicated in statin-induced myopathy [31]. These provide supportive, preliminary evidence for a role for membrane transporters in the cause for SAMs, like myositis and myalgia.

## 9. Future Implications of Statins

The distinction between cholesterol-dependent and -independent effects, and therefore, statin pleiotropy, is still contentious. Clinically, it can be difficult to separate cholesterol-lowering and non-cholesterol-lowering effects within clinical trials, whether the effects be harmful (i.e., myalgias) or beneficial (i.e., cardioprotective effects). One such hypothesis for the explanation of SAMs is that cholesterol-dependent effects are predicated on the reduction in cholesterol content in the skeletal myocyte sarcolemma, resulting in membrane frailty and possibly cytolysis [86,183]. However, hereditary diseases that impair cholesterol synthesis are not associated with myopathy [184]. Similarly, evidence of cholesterol-independent effects in statin usage is founded on the speed at which they act, as cholesterol-independent effects can appear within days [96]. SAMs can also be reversed upon statin discontinuation and reoccur with statin rechallenge [22]. This favours the hypothesis that statin pleiotropy does exist. Beneficial, off-target effects of statins are expansive, although not completely understood. They include improved endothelial function, increased plaque stability, antithrombotic and anti-inflammatory effects, reduced risk of dementia and Alzheimer disease, reduced ischaemic stroke, and in some cases, can offer benefit in the treatment of cancer [15,35,36,37,185].

It is believed that these positive effects are a result of the inhibition of the cholesterol biosynthesis pathway, and therefore, a lack of isoprenoid intermediate synthesis, like farnesylpyrophosphate (FPP) and geranylgeranylpyrophosphate (GGPP) (Figure 1) [186]. These are important in lipid attachment for posttranslational modification of cell-signalling proteins [96]. Downstream from the conversion of FPP to GGPP, RhoA is formed and is a potent inhibitor of endothelial nitric oxide synthase (eNOS); therefore, statin therapy might cause these eNOS inhibitory intermediates to not be formed. eNOS is a haem protein that offers many beneficial, cardioprotective effects, namely in the synthesis of nitric oxide upon the detection of shear stress within blood vessels [187,188,189]. Statins have similarly been shown to accelerate angiogenesis and reduce the severity of cerebral ischaemia in ischaemic strokes in mice [190,191,192]. eNOS localises within phospholipid membranes in the caveolin-1 membrane protein, and its association with membranes is facilitated by its palmitoylation. Palmitoylation is the covalent attachment of fatty acid residues on cysteine residues 15 and 26 of the eNOS protein. It has been demonstrated that lipophilic molecules, specifically the calcium channel blocker, amlodipine, are able to unclamp and release eNOS from caveolin-1 [193,194]. This insight has a possible two-fold application: (a) it offers a possible mechanism, independent of the cholesterol biosynthesis pathway, to increase eNOS release, and (b) provides a seminal insight into the possibility of harnessing statins as a lipophilic mechanism for eNOS upregulation and nitric oxide release. Evidence of statins increasing the activity of eNOS is not a novel discovery and has been shown to improve nitric oxide release and reduced free radical scavenging of nitric oxide [195]. Simvastatin was described to significantly enhance eNOS without changing its expression (i.e., without gene upregulation) possibly via phosphorylation by protein kinase B (Akt/PKB) and/or AMP-activated protein kinase (AMPK) [196]. Cerivastatin was seen to activate calcium-activated potassium channels in human endothelial cells, which also contributes to statin-induced nitric oxide production [197].

Despite the evidence of statin-induced pleiotropy, further evidence is still required to fully understand the molecular interactions statins have with membranes and in other biological processes. This is because studies investigating the effects of statins on membranes, and in cell culture, use statin concentrations in the micromolar range, far exceeding the clinically relevant serum concentration ~100 nM [198]. Utilisation of new models to assess statin membrane pleiotropy, and also, novel drugs to counteract statin pleiotropy, have been recently investigated. Here, tethered bilayer lipid membranes (tBLMs), which are artificial, mimetic membranes, are able to offer fast detection of biologically relevant effects of statins, and its results parallel those in biological assays [30].

It has been elucidated that an increase in statin hydrophilicity, and amine residues, namely cycloalkyl sulphonamide groups, increase the hepatoselectivity of statins to the HMGR binding site [137]. This would, in turn, reduce the non-selective, extra-hepatic binding that is hypothesised to result in SAMs. Alternatively, the discovery of an HMGR degrader, Cmpd 81, opens a new avenue in the treatment of hypercholesterolaemia. The reduction in cholesterol produced by statins can cause a brief upregulation of HMGR and a simultaneous reduction in its degradation. This can limit the effectiveness of statins, and result in increased dosages, which are associated with increased pleiotropy. Here, Cmpd 81 prevents HMGR accumulation, and reduces serum cholesterol levels. Therefore Cmpd 81, used in synergy with statins, could offer a novel way to treat hypercholesterolaemia, and reduce some of the off-target effects associated with statin administration [199].

## 10. Conclusions

In this review, we discuss the pleiotropic effects of statins with respect to the structural and biophysical properties of this drug class. We specifically focus on the property of lipophilicity and how this relates to target specificity, efficacy, and pleiotropy. The specific focus on the relationship between statin structure and pleiotropic effects provides new insights on this clinically important drug. Despite this, uncertainties in the cause of these prevalent side effects of statin usage still exist and suggest further research is needed to understand and eventually abolish the occurrence of statin-induced myopathies.
